# Takotsubo-Like Myocardial Dysfunction Accompanied with Cerebellar Hemorrhage

**DOI:** 10.1155/2012/306171

**Published:** 2012-08-02

**Authors:** Akira Tempaku, Tsugiyasu Kanda

**Affiliations:** ^1^Department of General Medicine, Kanazawa Medical University Himi Municipal Hospital, 31-9 Saiwai-cho, Toyama, Himi 935-8531, Japan; ^2^Department of Neurosurgery, Hokuto Hospital, 7-5 Inadacho-kisen, Hokkaido, Obihiro 080-0833, Japan

## Abstract

We report a 71-year-old woman with takotsubo-like myocardial dysfunction accompanied with cerebellar hemorrhage. On admission time, although she was unconscious by cerebellar hemorrhage, no obvious heart failure and serological disorder were observed. Three days later, operation for extraventricular drainage was performed. However, conscious level did not change. Four days after admission, the change of electrocardiogram wave pattern and the decrement of heart wall motion were detected. These findings revealed takotsubo-like myocardial dysfunction had occurred. Physical stresses by cerebellar hemorrhage and cranial operation might cause cardiac disorder. This is a remarkable case of takotsubo-like myocardial dysfunction, which is brought about cerebellar hemorrhage against subarachnoid hemorrhage.

## 1. Introduction

Transient left ventricular apical ballooning (TLVAB) characterizes takotsubo cardiomyopathy. It has been named after the shape of the heart that resembles Japanese octopus catcher pot with short narrow neck and round bottom [1–4]. This cardiomyopathy is diagnosed by the typical electrocardiogram pattern (ST segment elevation and giant negative T wave) and the abnormal left ventricular wall motion in cardioechogram [5, 6]. At first, takotsubo cardiomyopathy cases were reported from Japanese clinicians, mainly [1, 3, 4, 7, 8]. Therefore, many researchers supposed that takotsubo cardiomyopathy was an endemic disease in Japan. Later, several reports about this cardiomyopathy were published from worldwide areas [9–13]. Nowadays, this cardiomyopathy is familiar to the world. The diagnostic criteria for takotsubo cardiomyopathy were developed by the Research Committee of Idiopathic Cardiomyopathy [5]. Guidelines define the takotsubo cardiomyopathy as a disease that exhibits an acute left ventricular ballooning of unknown cause. Cerebrovascular patients, who have an apical systolic ballooning similar to that in takotsubo cardiomyopathy, are diagnosed as “cerebrovascular disease with takotsubo-like myocardial dysfunction.” Takotsubo-like myocardial dysfunction has been reported to accompany with subarachnoid hemorrhage (SAH) [14–19]. In addition, cerebral infarction has been related to this myocardial dysfunction, too [20–25]. However, few reports have been described to associated with intracerebral or cerebellar hemorrhage. We present here the cerebellar bleeding woman case accompanied with such myocardial dysfunction. This is a rare case report of takotsubo-like myocardial dysfunction which onset not after subarachnoid but after cerebellar hemorrhage. 

## 2. Case Presentation

**Figure 1:**
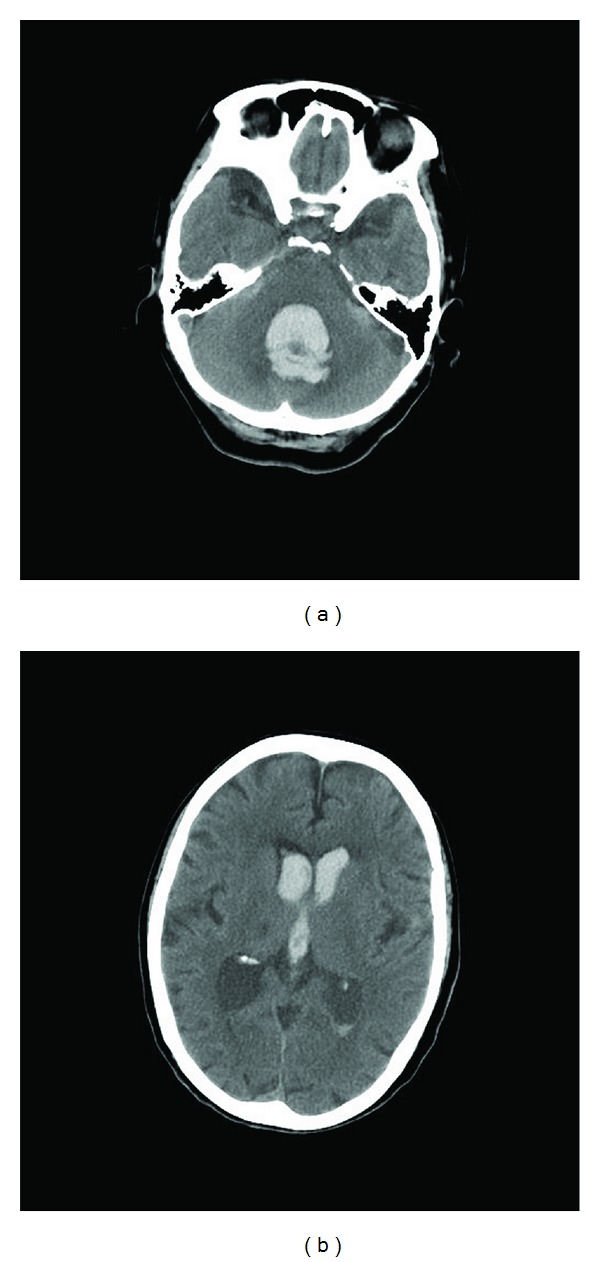
CT scan of the head was performed on admission day. Cerebellar hemorrhage in vermis (a) and intraventricular bleeding (b) were observed.

**Figure 2:**
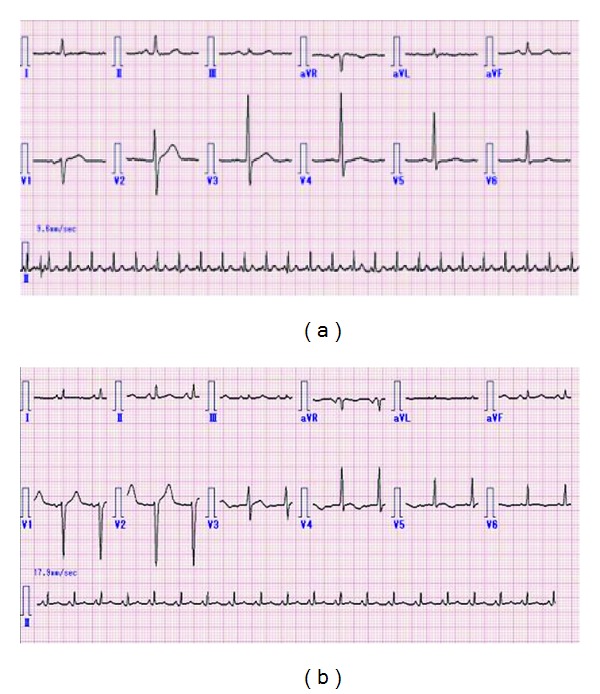
Electrocardiogram analyses were performed on admission day (a) and four days after admission (b).

A 71-year-old woman was admitted to Kanazawa Medical University Himi Municipal hospital due to loss of consciousness after sudden severe headaches and dizziness on June 4, 2011. The level of consciousness was that eye opening was nil (E1), best motor response was extended (M2), and best verbal response was nil (V1) in Glasgow Coma Scale. Hypertension (228/126 mmHg on admission time) was observed. Direct and indirect light reflexes were lost and both pupils were miotic (2 mm/2 mm). However, doll's eye sign was not observed. Brain-computed tomography (CT) showed midcerebellar hemorrhage with intraventricular bleeding (Figure 1). Following the intraventricular bleeding, ventricle moderately expanded. Electrocardiography on admission time was sinus rhythm. Further, no ST change was detected in any leads (Figure 2(a)). No obvious cardiac murmur was detected. Serological analysis showed almost normal level in inflammatory marker (C-reactive protein; CRP), cardiac enzymes (CK), other organ related enzymes (AST, ALT, LDH, and creatine), electrolytes, and other biochemical substances. Constitutional symptom was not operable. Conservative treatment with depressors and styptic was continued. Two days after admission, verbal impairment was slightly improved and simple word was heard. Extraventricular drainage was performed on June 6, 2011 (three days after admission). Drainage pressure was kept at 20 cm H_2_O. The discharge amount was about 150 mL per day. Although drainage was continued, no remarkable improvement of conscious level was observed. Four days after admission, electrocardiography showed negative T wave in leads V4, V5, and V6. Further, ST elevation was observed in leads V1, V2, and V3 (Figure 2(b)). Echocardiogram showed hypokinesis of wall motion and the apical ballooning. Left ventricular ejection fraction decreased to 41%. However, left ventricular diameter in end diastole was 42 mm which is within normal range. Any fluid in pericardium and thrombus in left ventricle were not detected. Takotsubo-like myocardial dysfunction with cerebrovascular disease was strongly suspected by these findings. Hypokinesis and low output of heart prevented us to perform the operation for hematoma depletion. During the medication, hematoma in midcerebellum gradually decreased. However, conscious level was not improved, anymore. After two weeks drainage, discharge amount decreased. Therefore, hydrostatic pressure for drainage was changed to 25 cm H_2_O. Nevertheless, no remarkable cerebrospinal fluid leakage was obtained. Drainage tube was removed on twenty days after admission (June 23, 2011). Twenty-two days after admission, systolic blood pressure became lower and reached 60 mmHg, rapidly. Oxygen saturation level in blood fell to 93%. In addition, heart rate gradually decreased. The patient died on that day (June 25, 2011) by cardiac dysfunction. 

## 3. Discussion

We diagnosed a 71-year-old woman with cerebellar hemorrhage accompanied with takotsubo-like myocardial dysfunction. The causes of such myocardial dysfunction are characterized by progression of catecholamine production and disturbance of autonomic nerve regulation [26–29]. High amount of catecholamine suppresses the motion of myocardium.

Cerebellar hemorrhage in vermis and intraventricular bleeding with hydrocephalus might cause physical stress to the patient. Further, operative treatment for extraventricular drainage also accumulated additional stress on her. These stresses might bring the autonomic imbalance, which is caused out of catecholamine regulation. Several stresses triggered takotsubo-like myocardial dysfunction. This myocardial dysfunction is usually associated with brain stroke [17]. Especially, SAH is frequently accompanied with this myocardial dysfunction [14–19]. The relation between cerebral infarction and takotsubo-like myocardial dysfunction will be gradually revealed by the later reports [20–25]. In contrast, a few researches point the correlation between cerebral hemorrhage and takotsubo-like myocardial dysfunction [30–33]. Much less, few reports show the association between cerebellar hemorrhage and takotsubo-like myocardial dysfunction [34]. This paper is a remarkable case of takotsubo-like myocardial dysfunction followed by cerebellar hemorrhage.

Cardiac beat is regulated by autonomic nerve control [35]. Sympathetic nervous system is controlled by several neurons from caeruleum nucleus, raphe nuclei, nuclei of solitary tract, and ventral nuclei of medulla oblongata. Parasympathetic nervous system is controlled by vagus nerve originated from dorsal nucleus of vagus and ambiguous nucleus. These autonomic nerve systems are regulated by hypothalamus. The paraventricular and other hypothalamic nuclei control preganglionic sympathetic and parasympathetic neurons. Autonomic nerve fibers pass through in periventricular zone and brain stem regions. Brainstem reticular formation exists in dorsal area of the brainstem. The cardiovascular center in medulla oblongata regulates heartbeat. Subarachnoid hemorrhage usually disturbs these autonomic nerve networks. As a result of such disturbance, high amount of noradrenalin is released. Catecholamine storm brings cardiac disorder known to takotsubo-like myocardial dysfunction [36, 37]. Noradrenalin causes microvascular spasm in heart mediating by  *α*2 receptors on myocardium [38]. In this case, cerebellar hemorrhage in vermis concomitant with intraventricular bleeding following ventricle enlargement caused damage to autonomic nuclei and nerve fibers. Because of cerebellar hemorrhage and ventricle enlargement, dorsal side of brainstem was easily damaged. As a result, reticular formation in brainstem was injured. These disorders might bring takotsubo-like myocardial dysfunction. 

In conclusion, not only subarachnoid but aslo cerebellar hemorrhage can cause to takotsubo-like myocardial dysfunction. The evaluation of cardiac function by ECG and echocardiogram is important to assess the onset of cardiomyopathy in patients with cerebellar hemorrhage.

## References

[1] Sato K, Masuda T, Kikuno T (1990). Left ventricular asynergy and myocardial necrosis accompanied by subarachnoid hemorrhage: contribution of neurogenic pulmonary edema. *Journal of Cardiology*.

[2] Sato H, Tateishi H, Uchida T, Kodama K, Haze K, Hon M (1990). Takotsubo-type cardiomyopathy due to multivessel spasm. *Clinical Aspect of Myocardial Injury: From Ischemia to Heart Failure*.

[3] Dote K, Sato H, Tateishi H, Uchida T, Ishihara M (1991). Myocardial stunning due to simultaneous multivessel coronary spasms: a review of 5 cases. *Journal of Cardiology*.

[4] Tsuchihashi K, Ueshima K, Uchida T (2001). Transient left ventricular apical ballooning without coronary artery stenosis: a novel heart syndrome mimicking acute myocardial infarction. *Journal of the American College of Cardiology*.

[5] Kawai S, Kitabatake A, Tomoike H (2007). Guidelines for diagnosis of takotsubo (Ampulla) cardiomyopathy. *Circulation Journal*.

[6] Sakamoto H, Nishimura H, Imataka K, Ieki K, Horie T, Fujii J (1996). Abnormal Q wave, ST-segment elevation, T-wave inversion, and widespread focal myocytolysis associated with subarachnoid hemorrhage. *Japanese Circulation Journal*.

[7] Kawai S, Suzuki H, Yamaguchi H (2000). Ampulla cardiomyopathy (“Takotusbo” cardiomyopathy)—reversible left ventricular dysfunction with ST segment elevation. *Japanese Circulation Journal*.

[8] Kurisu S, Sato H, Kawagoe T (2002). Tako-tsubo-like left ventricular dysfunction with ST-segment elevation: a novel cardiac syndrome mimicking acute myocardial infarction. *American Heart Journal*.

[9] Desmet WJR, Adriaenssens BFM, Dens JAY (2003). Apical ballooning of the left ventricle: first series in white patients. *Heart*.

[10] Seth PS, Aurigemma GP, Krasnow JM, Tighe DA, Untereker WJ, Meyer TE (2003). A syndrome of transient left ventricular apical wall motion abnormality in the absence of coronary disease: a perspective from the United States. *Cardiology*.

[11] Ibáñez B, Navarro F, Farré J (2004). Tako-tsubo transient left ventricular apical ballooning is associated with a left anterior descending coronary artery with a long course along the apical diaphragmatic surface of the left ventricle. *Revista Espanola de Cardiologia*.

[12] Connelly KA, MacIsaac AI, Jelinek VM (2004). Stress, myocardial infarction, and the “tako-tsubo” phenomenon. *Heart*.

[13] Osherov A, Matetzky S, Beinart R, Hod H (2004). Transient left ventricular apical ballooning (Tako-tsubo): the syndrome that mimics acute myocardial infarction. *The Israel Medical Association Journal*.

[14] Mayer SA, Fink ME, Homma S (1994). Cardiac injury associated with neurogenic pulmonary edema following subarachnoid hemorrhage. *Neurology*.

[15] Mayer SA, LiMandri G, Sherman D (1995). Electrocardiographic markers of abnormal left ventricular wall motion in acute subarachnoid hemorrhage. *Journal of Neurosurgery*.

[16] Ono Y, Kawamura T, Ito J, Kanayama S, Miura T, Kikuchi F (2004). Ampulla (Takotsubo) cardiamyopathy associated with subarachnoid hemorrhage worsening in the late phase of vasospasm—case report. *Neurologia Medico-Chirurgica*.

[17] Hessel EA (2006). The brain and the heart. *Anesthesia & Analgesia*.

[18] Lee VH, Connolly HM, Fulgham JR, Manno EM, Brown RD, Wijdicks EFM (2006). Tako-tsubo cardiomyopathy in aneurysmal subarachnoid hemorrhage: an underappreciated ventricular dysfunction. *Journal of Neurosurgery*.

[19] Cardin C, Roncalli J, Lairez O (2011). Subarachnoid haemorrhage associated with midventricular Tako-Tsubo syndrome. *International Journal of Cardiology*.

[20] Wang TD, Wu CC, Lee YT (1997). Myocardial stunning after cerebral infarction. *International Journal of Cardiology*.

[21] Sadamatsu K, Tashiro H, Maehira N, Yamamoto K (2000). Coronary microvascular abnormality in the reversible systolic dysfunction observed after noncardiac disease. *Japanese Circulation Journal*.

[22] Nakamura A, Asano M, Katagiri K (2003). A case report of ampulla-like shape left ventricular abnormality induced by the stress of cerebral infarction. *Shinzou*.

[23] Ueno Y, Inoue T, Shibazaki K (2006). A case of Takotsubo cardiomyopathy associated with embolic basilar artery occulusion. *Japanese Journal of Stroke*.

[24] Yoshimura S, Toyoda K, Ohara T (2008). Takotsubo cardiomyopathy in acute ischemic stroke. *Annals of Neurology*.

[25] Kato Y, Takeda H, Furuya D, Deguchi I, Tanahashi N (2009). Takotsubo cardiomyopathy and cerebral infarction. *Clinical Neurology*.

[26] Mori H, Ishikawa S, Kojima S (1993). Increased responsiveness of left ventricular apical myocardium to adrenergic stimuli. *Cardiovascular Research*.

[27] Shimizu M, Kawata M, Okada T (2001). A case of “Takotsubo” cardiomyopathy (ampulla cardiomyopathy) associated with elevated noradrenaline level in the acute phase. *Shinzou*.

[28] Ortak J, Kurowski V, Wiegand UKH (2005). Cardiac autonomic activity in patients with transient left ventricular apical ballooning. *Journal of the American College of Cardiology*.

[29] Burgdorf C, von Hof K, Schunkert H, Kurowski V (2008). Regional alterations in myocardial sympathetic innervation in patients with transient left-ventricular apical ballooning (Tako-Tsubo cardiomyopathy). *Journal of Nuclear Cardiology*.

[30] Deininger MH, Radicke D, Buttler J, Scheufler KM, Freiman T, Zentner JF (2006). Tako-tsubo cardiomyopathy: reversible heart failure with favorable outcome in patients with intracerebral hemorrhage. *Journal of Neurosurgery*.

[31] Izumi M, Watanabe H, Fujiwara R (2008). Clinical significance of electrocardiographic alternations in patients with intracranial hemorrhage. *Japanese Journal of Stroke*.

[32] Rahimi AR, Katayama M, Mills J (2008). Cerebral hemorrhage: precipitating event for a Tako-tsubo-like cardiomyopathy?. *Clinical Cardiology*.

[33] Izumi M, Ono Y, Sato M (2009). Successful percutaneous coronary intervention in patient with typical apical ballooning syndrome triggered by intracranial hemorrhage. *Shinzou*.

[34] Y-Hassan S, Lindroos M (2011). Cerebellar haemorrhage triggered Takotsubo-like left ventricular dysfunction syndrome. *International Journal of Cardiology*.

[35] Benarroch EE (1993). The central autonomic network: functional organization, dysfunction, and perspective. *Mayo Clinic Proceedings*.

[36] Meglič B, Kobal J, Osredkar J, Pogačnik T (2001). Autonomic nervous system function in patients with acute brainstem stroke. *Cerebrovascular Diseases*.

[37] Arab D, Yahia AM, Qureshi AI (2003). Cardiovascular manifestations of acute intracranial lesions: pathophysiology, manifestations, and treatment. *Journal of Intensive Care Medicine*.

[38] Heusch G, Baumgart D, Camici P (2000). *α*-Adrenergic coronary vasoconstriction and myocardial ischemia in humans. *Circulation*.

